# Supplementary material for: Comprehensive Annotation and Functional Exploration of MicroRNAs in Lettuce
Source: Front Plant Sci. 2021 Dec 24;12:781836. doi: 10.3389/fpls.2021.781836 (PMC8739914; doi:10.3389/fpls.2021.781836)
Supplement: Supplementary file 3 [file Data_Sheet_9.ZIP › GSM2230753.plot/Lsa-miRN55_Lsat_1_v5_gn_1_82220.1_645_TPlot.pdf]

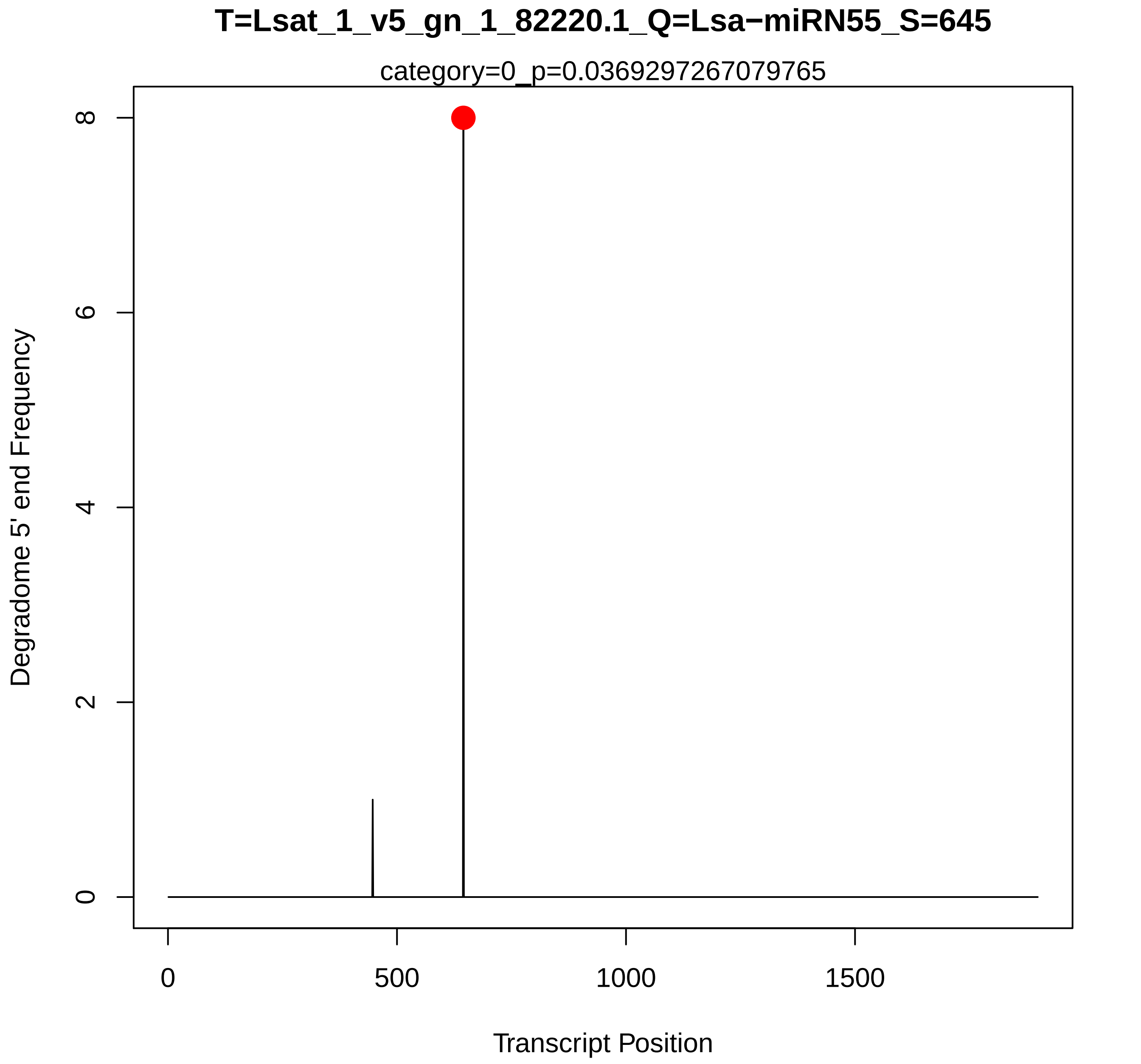

T=Lsat_1_v5_gn_1_82220.1_Q=Lsa−miRN55_S=645
category=0_p=0.0369297267079765
Degradome 5' end Frequency
0
2
4
6
8
0
500
1000
1500
Transcript Position
